# TLR3 deletion inhibits programmed necrosis of brain cells in neonatal mice with sevoflurane-induced cognitive dysfunction

**DOI:** 10.18632/aging.204092

**Published:** 2022-06-06

**Authors:** Qi Zhang, Yanan Li, Jiaxu Yu, Chunping Yin, Junfei Guo, Juan Zhao, Qiujun Wang

**Affiliations:** 1 Department of Anesthesiology, The Third Hospital of Hebei Medical University, Shijiazhuang 050051, Hebei, China; 2 Department of Anesthesiology, Children′s Hospital of Hebei Province Affiliated to Hebei Medical University, Shijiazhuang 050031, Hebei, China; 3 Department of Orthopedics, The Third Hospital of Hebei Medical University, Shijiazhuang 050051, Hebei, China; 4 Experimental Teaching Center of Hebei Medical University, Shijiazhuang 050017, Hebei, China

**Keywords:** TLR3, sevoflurane, cognitive dysfunction, programmed necrosis

## Abstract

This research aimed to explore the influence of TLR deletion on sevoflurane-induced postoperative cognitive dysfunction in neonatal mice. Herein, WT and TLR3 KO neonatal mice, each with 24, were randomly divided into control group, sevoflurane group, and TLR3^−/−^+sevoflurane group. The hippocampal neurons of WT, TLR3 KO and RIP3 KO neonatal mice in C group, SEV group, TLR3^−/−^+SEV group and RIP3^−/−^+SEV group were extracted for *in vitro* experiments. The results revealed the degeneration and necrosis of nerve cells in SEV group. Microscopic findings indicated that nerve cells showed shrinkage and nuclear hyperchromatism, along with lessening or even disappearance of nuclei and enlargement of cell spaces, and apoptotic cells in the brain tissues were evidently increased. Compared with SEV group, TLR3^−/−^+SEV group displayed reductions in these phenomena. Additionally, SEV group showed the reduced SHP2 expression and the increased expressions of proteins associated with TLR signaling pathway and apoptosis. Furthermore, there were no obvious differences in the expressions of such proteins in hippocampal neurons between RIP3^−/−^+SEV and TLR3^−/−^+SEV groups. The results confirmed that inhibiting RIP3 phosphorylation and suppressing TLR3 expressions exerted the same influence on the expressions of these proteins in the hippocampus of neonatal mice with sevoflurane-induced cognitive dysfunction. Based on these, it is speculated that TLR3 influences neonatal mice with sevoflurane-induced cognitive dysfunction probably by regulating RIP3 phosphorylation.

## INTRODUCTION

Postoperative cognitive dysfunction (POCD) is a common complication following anesthesia, which mainly involves memory impairment, abstract thinking disorder and disorientation, as well as the decline in ability to carry out social activities [1]. POCD is considered one of the postoperative complications in the central nervous system and belongs to a mild neurocognitive disorder [2]. Previous studies have revealed that the incidence of POCD within 7 days after non-cardiac surgery reached up to 9.1–17% [3, 4]. The pathogenesis of POCD involves the dysfunctions in the central nervous system, endocrine system and immune system, but its specific mechanism remains unclear [5]. The occurrence of POCD is related to many factors, including preoperative factors (such as age and physical condition), intraoperative factors (such as operation mode, operation time, anesthesia mode and intraoperative hypotension) and postoperative factors (such as infection). Among them, age and anesthesia are the most important. Most anesthetics have certain neurotoxicity, which can lead to some degree of neurological damage [6].

The existing view is that POCD is a neurological decline induced by surgery and anesthesia and influenced by the combination of multiple factors. At present, sevoflurane as an inhalational anesthetic is widely used in clinics [7], but it can cause damage to the development, learning and memory functions of the central nervous system. If sevoflurane is inhaled excessively, it will exacerbate endoplasmic reticulum stress and calcium overload and accelerate apoptosis of hippocampal neurons, eventually aggravating cognitive dysfunction in mice [8, 9]. Thus, there exist correlations between sevoflurane-induced POCD and neuronal apoptosis. The developing brain is in the stage of rapid development. At this stage, the development of the nervous system is not mature and is extremely sensitive to external environmental stimuli. Once exposed to neurotoxic substances, the development of the nervous system and cognitive function will be seriously affected. Premature and repeated exposure to sevoflurane has been proven to cause long-term impacts on the developing brain [10]. A clinical study also denoted that general anesthesia received for many times (≥2 times) or for a long time (>2 h) before the age of 3 markedly reduces problem processing speed and fine motor coordination ability in adolescence and adulthood [2]. However, the specific mechanism of sevoflurane-induced cognitive impairment in developing brain has not been fully clarified.

Medical bioinformatics is a cross-discipline subject aiming to store, retrieve, analyze and interpret biological and medical data based on computer science [11]. The rapid development of gene microarray and high-throughput sequencing technology in recent years has enabled researchers to quickly conduct the thorough analysis of transcriptomes and genomes, thus strongly driving the advancements of life sciences. In this research, differentially expressed genes (DEGs) were subjected to the analysis of the Kyoto Encyclopedia of Genes and Genomes (KEGG) pathway, and the KEGG pathway diagram was drawn to demonstrate the enrichment on the neurotrophin signaling pathway, toll-like receptor (TLR) signaling pathway, cell cycles, etc.

TLRs are highly conserved pattern recognition receptors and can transmit signals in innate immune response and inflammatory response. They function by detecting microorganisms in the external environment and then triggering the cascade of downstream signals [6]. Hippocampal neurogenesis involves multiple TLRs, which exert vital functions in the diseases related to cognitive function, emotion and memory impairment [10]. Among them, TLR3 specifically modulates the TIR-domain-containing adapter-inducing interferon-β (TRIF)-dependent signaling pathway and the activation of TLR3/TRIF signal participates in the occurrence and development of neuroinflammation [12]. Furthermore, TLR3 deletion has been uncovered to remarkably improve hippocampal-dependent memory and reduce anxiety-like behavior due to certain fears [13]. Nonetheless, it is still unclear whether TLR3 receptor is involved in sevoflurane-induced POCD in developing brain.

Hence, this study aimed to investigate the influence of TLR deletion on sevoflurane-induced POCD in neonatal mice through inducing POCD of neonatal mice by sevoflurane and then extracting their hippocampal neurons to conduct *in vivo* and *in vitro* experiments, so as to explore the potential molecular mechanisms behind the occurrence and development of POCD.

## MATERIALS AND METHODS

### Bioinformatics analysis

The dataset GSE95426 of POCD-related messenger ribonucleic acid (mRNA) gene expression was downloaded from the Gene Expression Omnibus (GEO) database (https://www.ncbi.nlm.nih.gov/gds/). Then the RNA-seq data of GSE95426 were subjected to quantile normalization using R language limma software package, followed by DEG analysis (|logFC|<1, *p* < 0.05). Moreover, R language software packages ggplot2 and pheatmap were used to draw the volcano plot and the cluster heatmap of DEGs.

### Functional enrichment analysis

Gene ontology (GO) and KEGG enrichment analyses were carried out for DEGs in the dataset GSE95426. The DAVID online database (https://david.ncifcrf.gov/) was adopted for DEG analysis at three levels of biological processes, cell components and molecular functions to integrate GO terms and create the biological process network of DEGs. Then the GO pathway diagram of DEGs and the enrichment analysis diagram of the KEGG pathway were drawn with GOplot and ggplot2 packages in the R language environment.

### Protein-protein interaction (PPI) network analysis and target gene screening of DEGs

Analysis of DEGs was conducted through PPI networks. Specifically, after DEGs were input into the online tool STRING, the interaction proteins were screened by the mobile cloud computing (MCC)-based topology analysis algorithm. The PPI results obtained were input into Cytoscape. Then using the Cytohubba plug-in, the target genes scoring top 10 were identified according to the degree algorithm.

### Gene set enrichment analysis (GSEA)

GSEA was performed for dataset and the GSEA-related pathway diagram was plotted.

### Experimental animals and model preparation

Herein, wild-type (WT, 6–9 weeks of age, weighing 20 ± 2 g, purchased from Beijing Vital River Laboratory Animal Technology Co., Ltd.) and TLR3 knockout (KO, 6–9 weeks of age, weighing 20 ± 2 g, purchased from Cyagen Biotechnology Co., Ltd.) neonatal mice, each with 24, were randomly classified into control group (C group), the sevoflurane group (SEV group), and TLR3^−/−^+sevoflurane group (TLR3^−/−^+SEV group). The animal use protocol was approved by the Animal Review Committee of the Third Hospital of Hebei Medical University (Code of Ethics: 2017-026-1). To induce general anesthesia of neonatal mice, both maternal and neonatal mice were placed in an acrylic anesthesia room with two ports, one connecting to a sevoflurane vaporizer (Drager, Germany) and the other to a multi-gas monitor (Datex-Ohmeda, USA). Mice were anesthetized on postnatal days 6, 7, and 8 or with 3% sevoflurane plus 60% oxygen for 2 h daily for three days in SEV and TLR3^−/−^+SEV groups. The C group was exposed to 30% humidified oxygen (N_2_ balance) in the sevoflurane-free acrylic anesthesia room.

### Cell culture and processing

The hippocampal neurons were extracted from WT, TLR3 KO (6–8 weeks of age, purchased from Cyagen Biotechnology Co., Ltd.) and receptor-interacting protein 3 (RIP3) KO neonatal mice (Born within 24-hours) for *in vitro* experiments. Firstly, 1-day-old neonatal mice were placed in precooling equilibrium solution and decapitated under deep anesthesia, and brain tissues were taken and placed in a petri dish containing ice-cold DMED/F12. Next, hippocampal tissues were separated under an anatomical microscope, cut into fragments (1 mm × 1 mm × 1 mm), and transferred into a centrifuge tube. Subsequently, an equal volume of 0.25% trypsin (HyClone, USA) was added and the tissues were placed in a CO_2_ incubator at a constant temperature of 37°C, followed by shaking the centrifuge tube once every 3 min. After digestion for 10 min, an equal volume of culture solution [DMEM culture medium + 20% fetal bovine serum (Gibco, USA)] was added to terminate the trypsin reaction. Following centrifugation, the supernatant was removed, the inoculum was added, and the cell suspension was filtered and collected. The morphology of neurons was observed under the inverted microscope (EVOS M7000 Imaging System, Thermo Fisher Scientific, USA). Neurons that had plump cell bodies and whose processes were densely connected into a network were included in this study. Dissociated hippocampal neurons were inoculated in a culture dish pre-coated with 5 μg/mL poly-L-lysine at a density of 5 × 10^5^/mL, 2,000 μL in each well. Twenty-four hours later, the special medium was replaced for hippocampal neurons [neurobasal + 3% B27] and the medium was replaced. On the 7th day of culture, microtubule-associated protein P2 and DAPI fluorescent staining were used to identify neurons. The neurons that had plump cell bodies, mature growth and purity >95% and whose processes were densely connected into a network were selected for later experiments.

Each group of culture dishes was placed in an anesthesia induction room at 37°C, with fresh air supply (21% O_2_, 5% CO_2_ and 69% N_2_) for C group or 5 h of additional 3.4% sevoflurane for SEV, TLR3^−/−^+SEV and RIP3^−/−^+SEV groups. Sevoflurane was stably maintained at 3.4% with a gas flow rate of 1 L/min and measured using an anesthesia monitor (Datex-Ohmeda, USA).

### Morris water maze

All mice were evaluated for cognitive abilities in the Morris Water Maze (MWM) test, during which each mouse was placed in the pool and then allowed to search for the platform. Specifically, the mice were slowly put into the water from each quadrant facing the pool wall, and the time required by the mice from putting into the water to climbing the hidden platform was recorded (i.e., escape latency). If the platform was not found within 60 s, the mice were guided to the platform for 20 s, and the escape latency was recorded as 60 s. A spatial probe test was conducted on the second day after the place navigation test was completed. Mice were dropped from the opposite quadrant into water twice and allowed to explore freely for 120 s. The video tracking system tracked and recorded the number of times the mouse crossing the hidden platform. At the end of each test, the mouse was dried in a cage under a heat lamp for 1–2 min, and returned to its normal cage.

### Nissl staining

Paraffin sections were deparaffinized with xylene, dehydrated by gradient alcohol and stained with 1% cresyl violet at room temperature for 20 min. After that, the sections were immersed and washed once in distilled water, followed by color separation with 70% alcohol. Then they were immersed in gradient alcohol and xylene separately for 2 min. Under a light microscope (BX51; Olympus, Tokyo, Japan), the total Nissl body count in the hippocampal CA1 region was analyzed by a pathologist blinded to grouping (three sections per slide).

### Detection of cell apoptosis by terminal deoxynucleotidyl transferase-mediated dUTP nick end labeling (TUNEL) assay

According to the instructions of the TUNEL kit, the specimens were permeabilized with 0.1% Trition X-100 and added with 200 μL of 3% H_2_O_2_. After rinsing three times with phosphate buffer saline (PBS), proteinase K was added to inactivate DNA and RNA, and the specimens were incubated in an incubator for 60 min in the dark. Then they were rinsed three times with PBS and stained with DAPI solution for 20 min. Finally, the specimens were washed and mounted. ImageJ was used to count the number of apoptotic cells under a fluorescence microscope. Apoptosis index (AI) = (the number of positive cells in each field)/(the total number of cells in each field) × 100%.

### Hematoxylin and eosin (H&E) staining

The sections were soaked in xylene overnight at room temperature and then dewaxed using gradient ethanol to water. Next, the sections were stained with hematoxylin for 3 min for nuclear staining, washed with clean water, and differentiated with 10% hydrochloric acid alcohol for 5 s. After washing with clean water, the sections were treated with 1% ammonia water for 15 s to return to blue and washed with clean water. Thereafter, the sections were stained with eosin for 1 min, conventionally dehydrated and sealed with environmental protection gum.

### Ultrastructure of hippocampal neurons

After hippocampus (approximately 1 mm × 1 mm × 3 mm) was collected and fixed with 4% glutaraldehyde and 1% osmium tetroxide, a series of ethanol solutions were used for dehydration of tissues, which were then embedded in epoxy, and double stained with uranyl acetate as well as with lead citrate. The ultrastructure of hippocampal neurons was observed by transmission electron microscopy (TEM) (H-7500; Hitachi, Japan).

### Western blotting

The brain hippocampal tissues and neurons of neonatal mice were collected and lysed with RIPA buffer containing protein phosphatase inhibitor (Sigma-Aldrich). Total proteins were extracted and transferred onto a PVDF membrane. Then the membrane was routinely blocked for 2 h and incubated with primary antibodies against TLR3, TRIF, Src homology region 2 domain-containing phosphatase-2 (SHP2), phosphorylated RIP3 (p-RIP3), calcium/calmodulin-dependent protein kinase II (CaMKII), nucleotide-binding oligomerization domain-like receptor protein 3 (NLRP3), gasdermin D (GSDMD), cysteinyl aspartate specific proteinase (Caspase)-1, Caspase-4, Caspase-11 and glyceraldehyde-3-phosphate dehydrogenase (GAPDH) and secondary antibodies. In this study, GAPDH was used as the internal reference, and bands were visualized with hypersensitive chemiluminescent liquid using LAS 4000 Imaging Analyzer (FujiFilm, Tokyo, Japan). ImageJ (NIH, Bethesda, MD, USA) was applied to analyze the relative intensities of individual bands.

### Statistical analysis

The data were analyzed by GraphPad Prism 7.0 and expressed as mean ± standard deviation. The differences between two groups were analyzed using Student’s *t*-test, and the differences among groups were analyzed by one-way ANOVA. *P* < 0.05 was considered statistically significant.

### Research ethics

The animal use protocol for this study has been reviewed and approved by Animal Review Committee of the Third Hospital of Hebei Medical University (Code of Ethics: 2017-026-1).

## RESULTS

### Screening of DEGs

**Figure 1:**
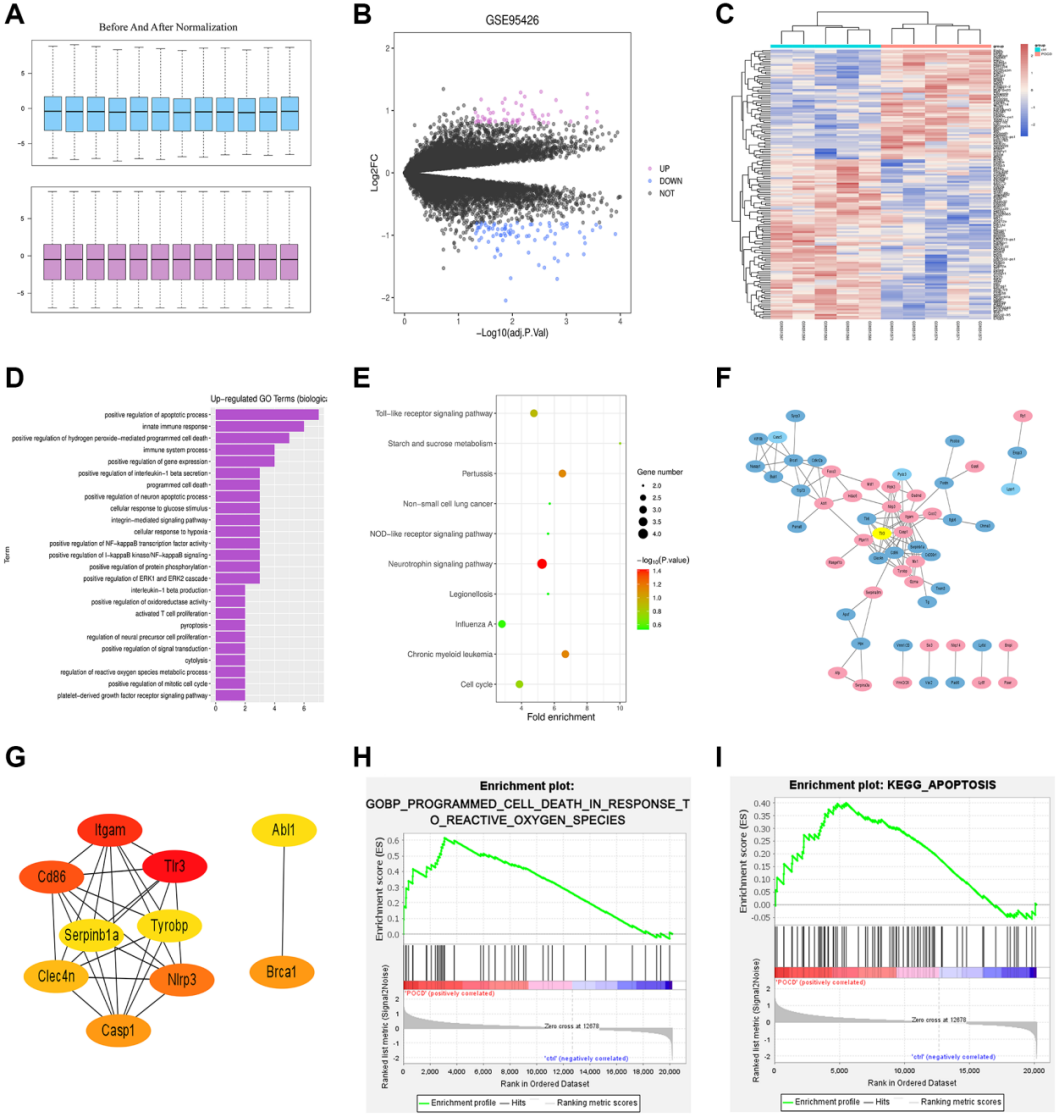
**Results of bioinformatics analysis.** (**A**) Before and after quantile normalization. (**B**) Volcano plot of visually grouped DEGs in the GSE75241 dataset. (**C**) Cluster heatmap of DEGs. (**D**) Up-regulating GO pathway diagram. (**E**) KEGG pathway diagram. (**F**) PPI network with 107 sides. (**G**) Key genes ranking top 10 in the PPI network. (**H**) and (**I**) GSEA-related pathway diagrams.

The POCD-related dataset GSE95426 was retrieved in the GEO database with the keyword “POCD” and then downloaded, followed by quantile normalization of the data. All the processed samples shared the same value, but the original gene order was reserved (Figure 1A). Subsequently, a total of 151 DEGs (including 62 up-regulated DEGs and 89 down-regulated ones) were screened out in the mRNAs of POCD from GSE95426 according to the criteria of |logFC|>1 and *p* < 0.05. Next, the volcano plot (Figure 1B) of visually grouped DEGs in the GSE75241 dataset was established using R software package ggplot2, where the up-regulated genes were denoted with red color and the down-regulated ones with blue color. Furthermore, the cluster heatmap (Figure 1C) of DEGs was drawn via R language package pheatmap. The cluster heatmap could measure the similarities between samples or gene expressions. In the aforementioned cluster heatmap, the X-coordinate represented sample clustering and each column stood for one sample. The clustering was performed based on the similarities of gene expressions between samples, that is, the more approximate the gene expressions between samples were, the closer they were to each other.

### Bioinformatics analysis

GO and KEGG enrichment analyses were performed for DEGs in the GSE95426 dataset. The DAVID online database (https://david.ncifcrf.gov/) was used for analysis of DEGs at the level of biological processes to integrate GO terms and create the biological process network of DEGs. Afterwards, the up-regulating GO pathway diagram (Figure 1D) of DEGs was drawn using R language, including up-regulating pathways like the positive regulation of apoptosis process, innate immune response and positive regulation of peroxide-mediated programmed cell death (PCD). Besides, the DEGs were subjected to the KEGG pathway analysis and the KEGG pathway diagram (Figure 1E) was drawn, which indicated the enrichment on the neurotrophin signaling pathway, TLR signaling pathway, cell cycles, etc.

### Establishment of PPI network for DEGs

The PPI between DEGs was predicted via STRING online software. The relevant data were downloaded and imported into Cytoscape to generate a PPI network with 107 sides (Figure 1F). Then using the Cytohubba plug-in, the key genes ranking top 10 were screened out according to the MCC-based topology analysis algorithm (Figure 1G).

### GSEA

GSEA was conducted for all genes using GSEA software (https://www.gsea-msigdb.org), and GSEA-related pathway diagrams (Figure 1H and Figure 1I) were drawn, which manifested the enrichment on the reactive oxygen species (ROS)-induced POCD and apoptosis pathways.

### Changes in cognitive function of neonatal mice in each group

**Figure 2:**
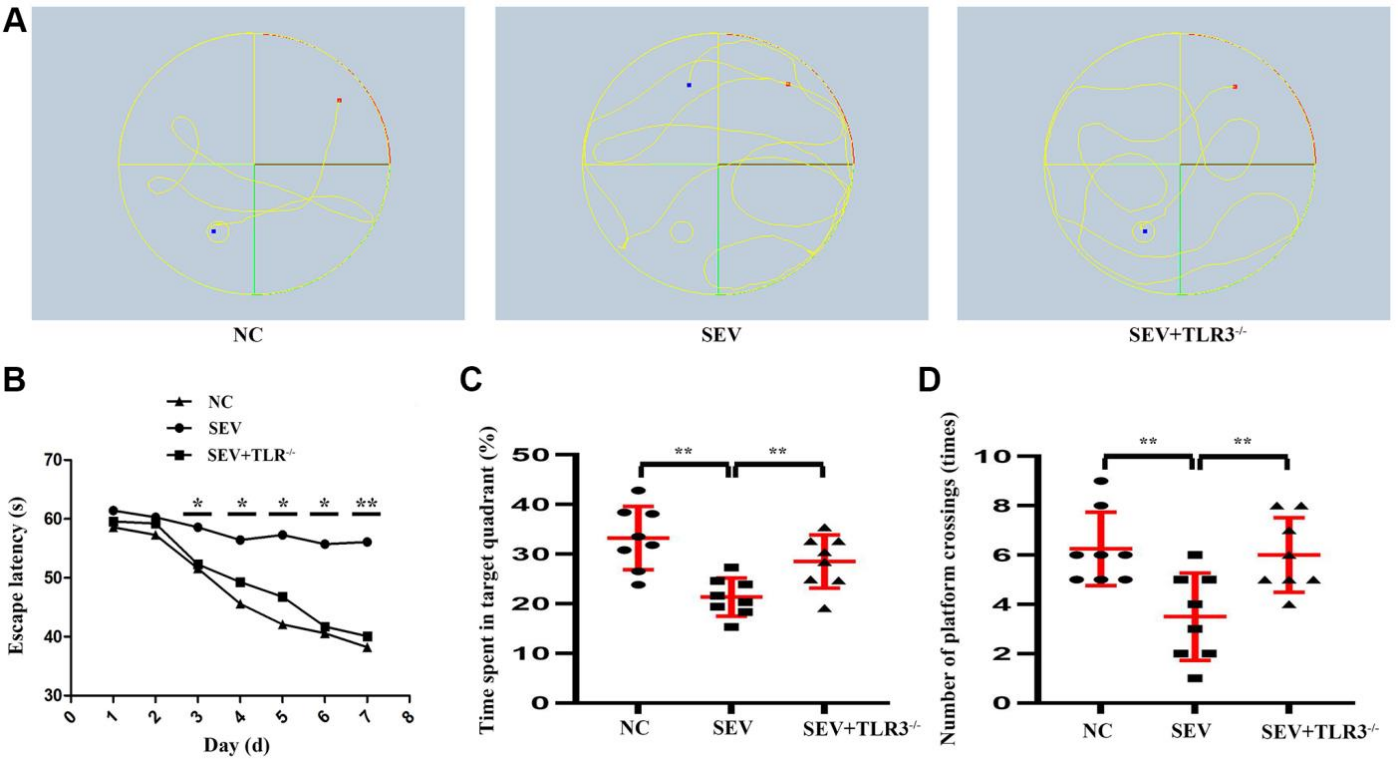
**Changes in cognitive function of neonatal mice in each group.** (**A**) Water maze movement track in mice. (**B**) Escape latency(s) of neonatal mice. (**C**) Time spent in target quadrant of neonatal mice. (**D**) Number of platform crossing (times) of neonatal mice.

Morris Water Maze test was used to detect the changes in cognitive ability of neonatal mice. The results revealed that during the navigation test, escape latency showed no difference in neonatal mice among the three groups on the 30th and 31st days after birth. From the following 3 to 7 days, SEV group exhibited a longer escape latency compared with control group. However, this increase was significantly reduced in TLR3^−/−^+SEV group, suggesting that sevoflurane caused spatial learning damage that was rescued by TLR3 deletion in neonatal mice. The results of the probe test revealed that the decrease in the number of platform crossings and time spent in the target quadrant in SEV group were significantly improved in TLR3^−/−^+SEV group, indicating that memory impairments after sevoflurane can be attenuated by TLR3 deletion in neonatal mice (Figure 2).

### Pathological changes in the hippocampal CA region of neonatal mice

**Figure 3:**
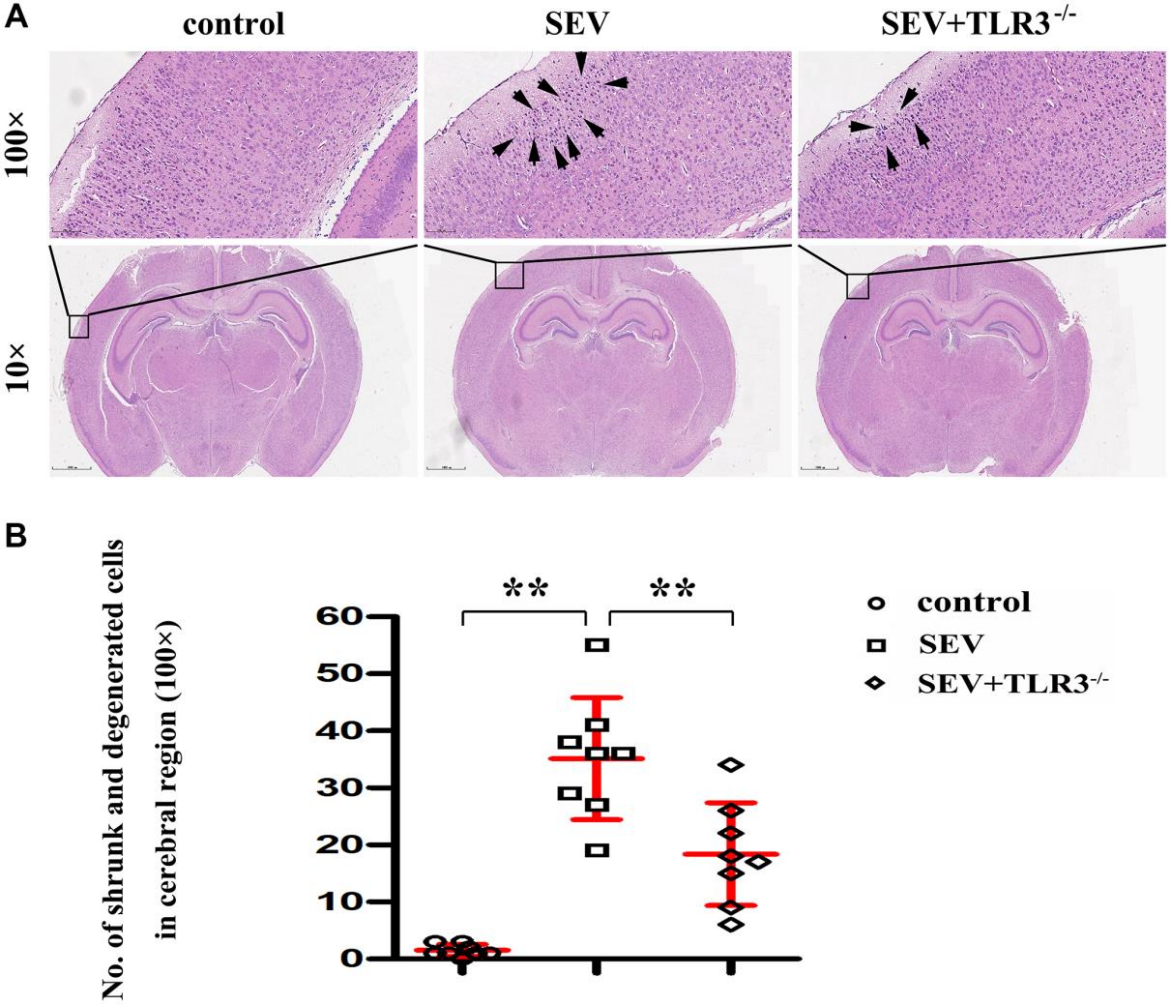
(**A**) Pathological changes (HE staining) in the cortical and hippocampal regions of neonatal mice (Magnification is 100× and 400×, respectively), (**B**) Statistical chart of pathological changes in cortical regions.

Results of HE staining were used to evaluate the suggested that neurons of brain cortical regions were arranged neatly, with normal morphology, clear boundary, round nucleus and obvious nucleolus in C group. Neurons of cortical regions were disorderly arranged, shrunk and degenerated, the nuclear boundary was unclear, the nucleolus disappeared, and there was interstitial edema in SEV group. The degree of atrophy and degeneration of neurons and interstitial edema in cortical regions in TLR3^−/−^+SEV group were less than that in SEV group, and the statistical icon were shown in Figure 3.

**Figure 4:**
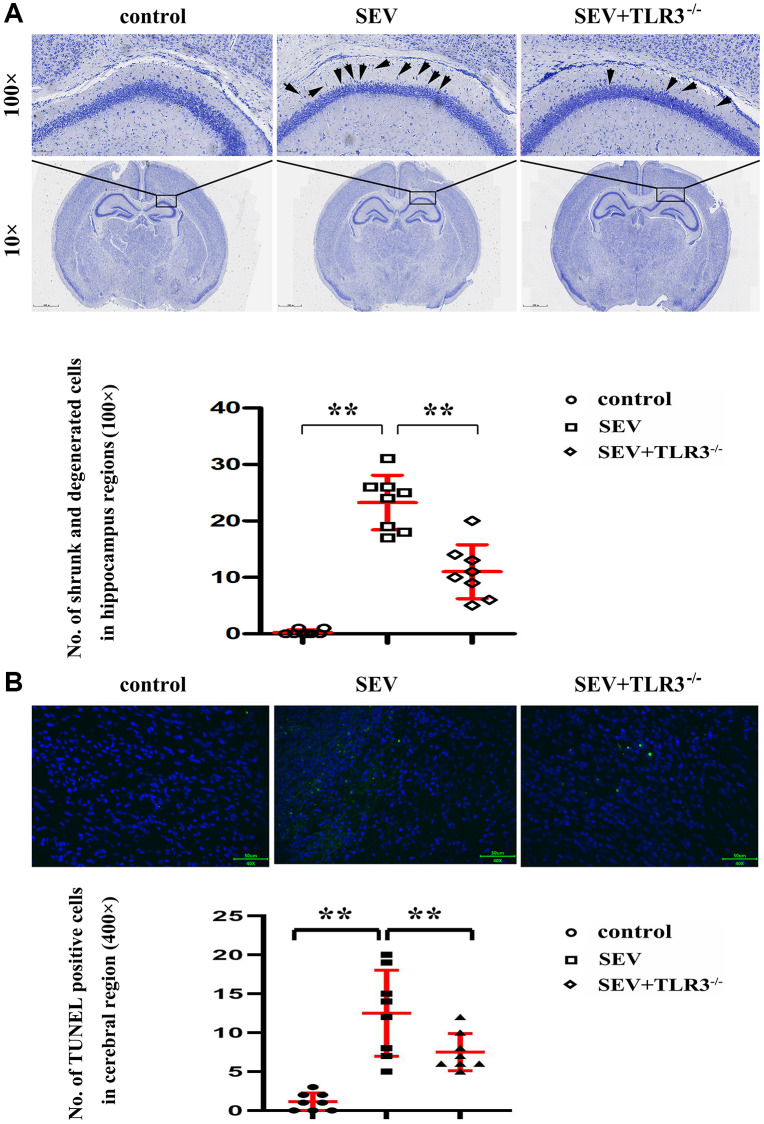
(**A**) Results of Nissl staining in the cortical and hippocampal regions of neonatal mice (Magnification is 100× and 400×, respectively), (**B**) TUNEL staining in the brain tissues of neonatal mice.

Nissl staining (Figure 4A) is better to manifest the following pathological changes in the hippocampal CA region of the mice in all groups: the hippocampal neurons of the neonatal mice in C group were morphologically normal, well-ordered, round and clear, with obvious nucleolus. The neurons in SEV group experienced degeneration and necrosis, and microscopic findings indicated that nerve cells showed shrinkage and nuclear hyperchromatism, along with lessening or even disappearance of nuclei and enlargement of cell spaces. In TLR3^−/−^+SEV group, the phenomena of neuron atrophy and nuclear lysis in neonatal mice were reduced in comparison with those in SEV group and the statistical icon were shown in Figure 4B.

### TLR3 deletion reduced cell apoptosis in brain tissues of neonatal mice with sevoflurane-induced cognitive dysfunction

The brain cell apoptosis index was detected by TUNEL staining method. The TUNEL-positive cell counts in different groups were shown in the histogram. Compared with C group and TLR3^−/−^+SEV group, the apoptotic cells in the brain tissues of neonatal mice were obviously increased in SEV group, indicating that TLR3 deletion reduces the cell apoptosis in the brain tissues of neonatal mice with sevoflurane-induced cognitive dysfunction (Figure 4B).

### TLR3 deletion alleviated sevoflurane-induced ultrastructural changes in hippocampal neurons of neonatal mice

**Figure 5:**
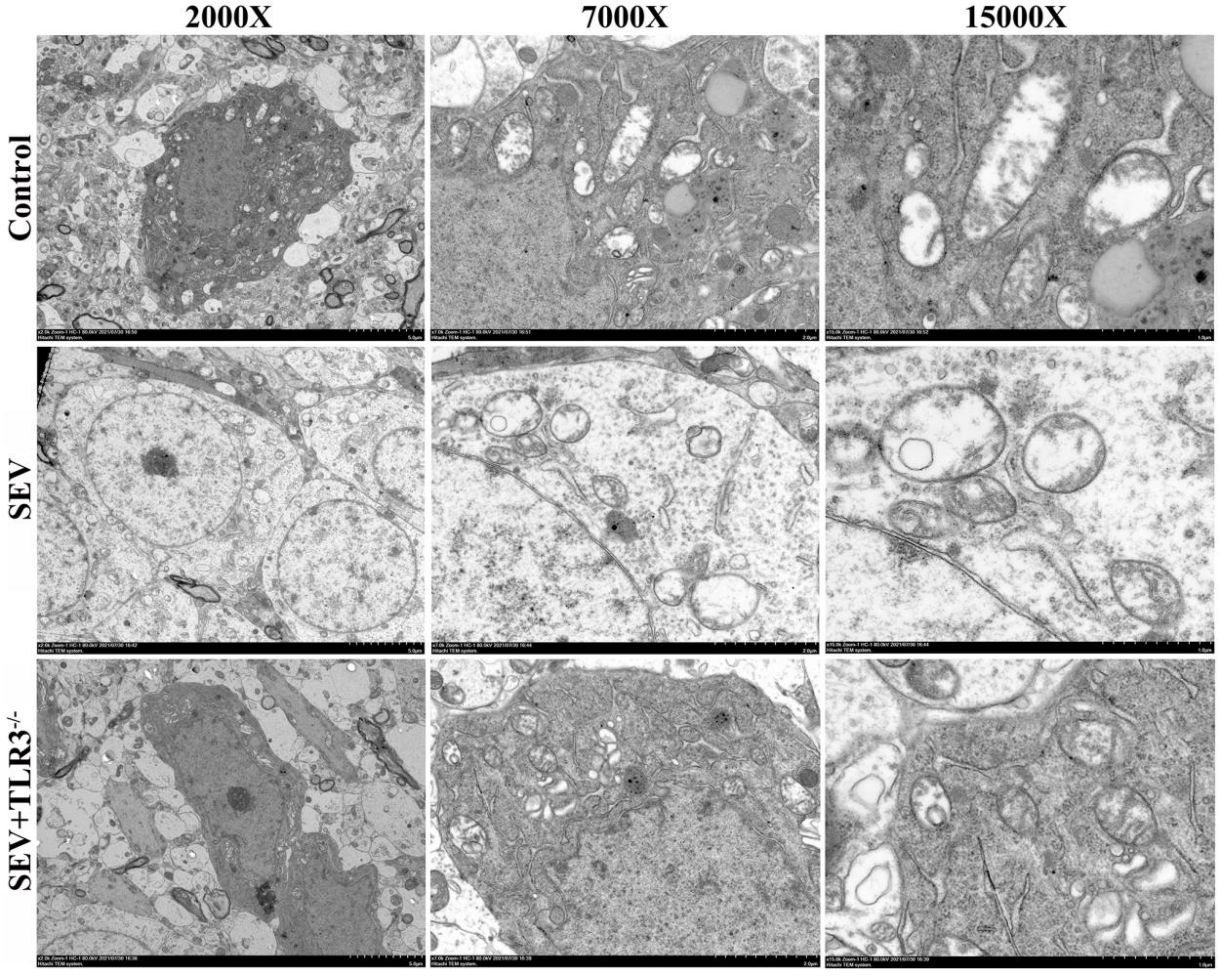
Representative transmission electron microscopy images of hippocampus of neonatal mice (Magnification is 2000×, 7000× and 15000×, respectively).

TEM maximumly provided the ability to observe the subcellular organelle in mouse brain tissue. The results showed that in C group, the mitochondria were oval or rod-shaped, surrounded by double membranes, and the inner membrane protruded inward into a flat ridge. The ridge was perpendicular to the long axis of mitochondria, with a large number of mitochondria. Compared with control group, the brain cells in SEV group had a large number of heterotypic mitochondria, which showed vacuolization, swelling, reduced matrix density and broken mitochondrial cristae. The inner and outer membrane defects of some severely damaged mitochondria and the cristae disappeared completely. Although TLR3^−/−^+SEV group still had a certain degree of injury, the degree of injury was less than that in SEV group (Figure 5).

### TLR3 deletion inhibited activation of TRIF and degradation of SHP2 and down-regulated expressions of TLR signaling pathway-related proteins

**Figure 6:**
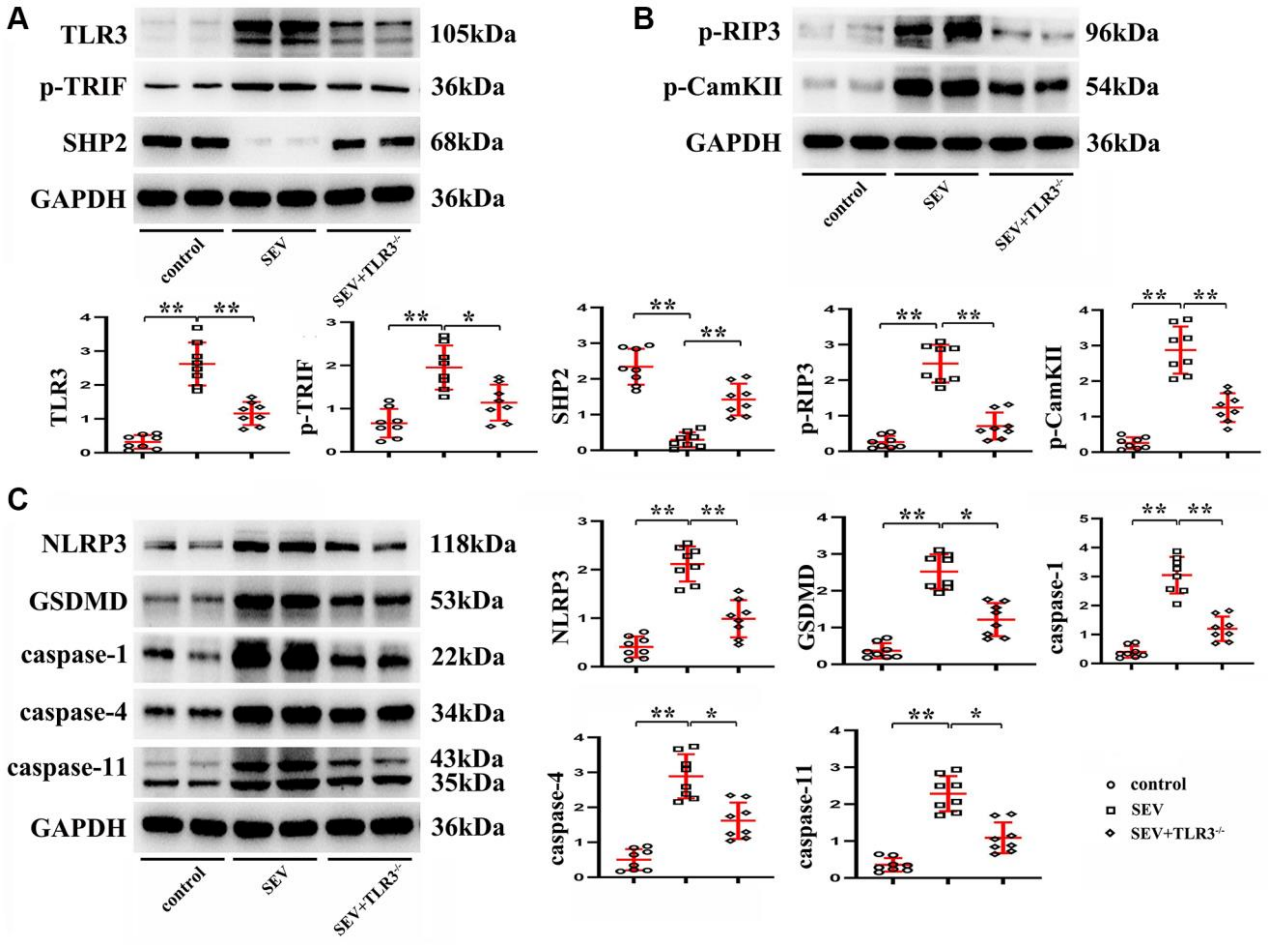
**TLR3 deletion inhibited activation of TRIF and degradation of SHP2 and down-regulated expressions of TLR signaling pathway-related proteins.** (**A**) Expressions of TLR3, p-TRIF and SHP2 in the hippocampus of neonatal mice. (**B**) Expressions of p-RIP3 and CaMKII in the hippocampus of neonatal mice. (**C**) Expressions of NLRP3, GSDMD, Caspase-1, Caspase-4 and Caspase-11 in the hippocampus of neonatal mice. ^**^p < 0.01, and ^*^p < 0.05.

Western blotting results manifested that in comparison with C group, the expression of SHP2 protein was significantly reduced, while those of TLR3, TRIF, p-RIP3, CaMKII, NLRP3, GSDMD, Caspase-1, Caspase-4 and Caspase-11 in the hippocampus of neonatal mice were evidently elevated in SEV group. The aforementioned protein expressions showed no obvious differences in the hippocampus of neonatal mice between C group and TLR3^−/−^+SEV group, indicating that TLR3 deletion is capable of repressing the activation of TRIF and the degradation of SHP2 and recovering the expressions of TLR signaling pathway-related proteins in the hippocampus of neonatal mice with sevoflurane-induced cognitive dysfunction (Figure 6).

### TLR3 deletion inhibited RIP3 phosphorylation and reduced programmed necrosis of hippocampal neurons in neonatal mice with sevoflurane-induced cognitive dysfunction by regulating TRIF and SHP2 expressions

**Figure 7:**
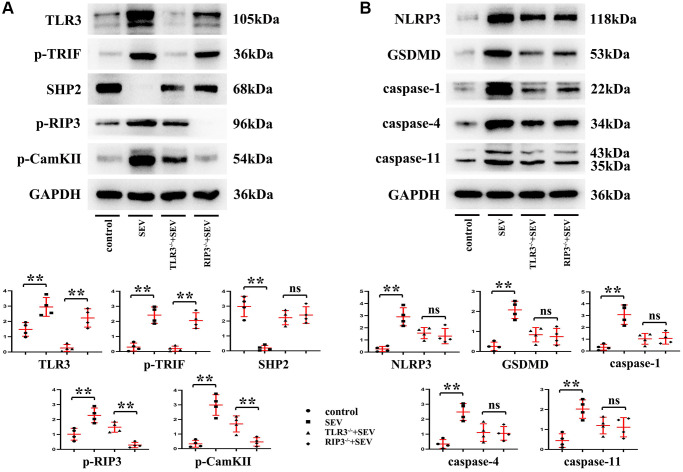
**TLR3 deletion inhibited RIP3 phosphorylation and reduced programmed necrosis of hippocampal neurons by regulating TRIF and SHP2 expressions.** (**A**) Expressions of TLR3, p-TRIF, SHP2, p-RIP3 and CaMKII in the hippocampal neurons. (**B**) Expressions of NLRP3, GSDMD, Caspase-1, Caspase-4 and Caspase-11 in the hippocampal neurons. ^**^p < 0.01.

The results of *in vitro* experiments revealed that relative to C group, the expression of SHP2 protein was evidently reduced, while those of TLR3, TRIF, p-RIP3, CaMKII, NLRP3, GSDMD, Caspase-1, Caspase-4 and Caspase-11 were apparently elevated in the hippocampal neurons of the neonatal mice in SEV group, but the expressions of these proteins were reversed in TLR3^−/−^+SEV group. Meanwhile, there were no obvious differences in the expressions of SHP2, p-RIP3, CaMKII, NLRP3, GSDMD, Caspase-1, Caspase-4 and Caspase-11 between RIP3^−/−^+SEV group and TLR3^−/−^+SEV group, meaning that the expressions of these proteins in the hippocampus of neonatal mice with sevoflurane-induced cognitive dysfunction can be influenced equally by inhibiting the phosphorylation of RIP3 and repressing the expression of TLR3, these results were shown in Figure 7A and 7B. The results hinted that TLR3 influences neonatal mice with sevoflurane-induced cognitive dysfunction probably by regulating RIP3 phosphorylation.

## DISCUSSION

POCD is a common complication after general anesthesia. The high risk of POCD triggered by inhalation anesthesia requires prompt prevention and treatment [14]. Sevoflurane is the most commonly used halogenated inhalational anesthetic for infants and young children. Animal experiments and retrospective clinical studies have demonstrated that sevoflurane anesthesia received for a long time or for many times during development is closely related to the long-term cognitive decline, which seriously affects the quality of life of infants and young children far away from their families [15]. Several studies have proven that sevoflurane can aggravate the cognitive dysfunction of rodents and induce neuroinflammation and hippocampal cell apoptosis in rats [16]. Moreover, the repeat inhalation of sevoflurane in neonatal stage will affect the voltage-gated channels in hippocampal CA1 pyramidal neurons [14] and also suppress these channels in the process of brain development, but its pathogenic mechanism remains elusive. In this study, TLR3 deletion was discovered to ameliorate repeat sevoflurane exposure-induced cognitive impairment in neonatal mice, inhibit RIP3 phosphorylation and reduce the programmed necrosis of hippocampal neurons.

Medical bioinformatics is a cross-discipline subject aiming to store, retrieve, analyze and interpret biological and medical data by means of computer science [17]. According to the bioinformatics-based KEGG pathway analysis of DEGs and the KEGG pathway diagram drawn in this study, the DEGs were enriched in the neurotrophin signaling pathway, TLR signaling pathway, cell cycles, etc.

The TLR family plays a significant role in maintaining the innate immunity of living organisms, withstanding the inflammatory reaction of pathogenic bacteria and keeping the balance between the protection and impairment of living organisms by immunologic processes [18, 19] and also acts as a crucial regulator in hippocampal neurogenesis. Multiple TLRs participate in hippocampal neurogenesis and exert vital functions in the diseases related to cognitive function, emotion and memory impairment. TLR3 specifically mediates the TRIF-dependent signaling pathway [12], and the activation of TLR3/TRIF may take part in the genesis and development of neuroinflammation [20]. In this study, POCD was induced in neonatal mice using sevoflurane and their hippocampal neurons were extracted for *in vivo* and *in vitro* experiments. In addition, the potential molecular mechanisms of TLR deletion for the genesis and development of POCD in neonatal mice were explored. The results revealed that TLR3 and TRIF were highly expressed while SHP2 was lowly expressed in the hippocampus of neonatal mice in SEV group. However, the situation was just the opposite in TLR3^−/−^+SEV group. Compared with SEV group, the positive cell counts of both TLR3 and TRIF were obviously reduced while that of SHP2 was evidently increased in the hippocampus of neonatal mice in TLR3^−/−^+SEV group, which were consistent with the results of Western blotting. Based on these, TLR3 deletion can lower the expression of TRIF and inhibit the degradation of SHP2. Furthermore, TLR3 deletion reduced the expressions of p-RIP3 and CaMKII in the hippocampal tissues and cells of the neonatal mice in SEA group. However, the expression of CaMKII was increased after sevoflurane-induced POCD, which would result in the calcium depletion of endoplasmic reticulum and then cell death [21, 22].

Evidence suggested that when human body is exposed to sevoflurane, NLRP3 can recruit Caspase-1 to form NLRP3 inflammasome and further induce the activation of Caspase-3 [23]. The persistent and excessive inflammation is crucial in the pathophysiologic mechanism of sevoflurane aesthesia-induced neurological dysfunction [24]. The sevoflurane-induced neuroinflammation can be ameliorated by repressing the activation of NLRP3, which is related to the sevoflurane-triggered pathological damage [25]. In this study, compared with C group, the expressions of NLRP3, GSDMD and Caspase-1 presented the consistent variation trends with the expressions of TRIF, p-RIP3 and CaMKII in the hippocampal tissues and neurons of neonatal mice in SEV group. The expressions of these proteins in TLR3^−/−^+SEV group were not obviously different from those in C group, indicating that TLR3 deletion can inhibit the activation of NLRP3 and further reduce the programmed necrosis of hippocampal tissues and neurons in neonatal mice with sevoflurane-induced cognitive dysfunction. The above conclusion was also verified by the TUNEL assay results. In comparison with SEV group, the apoptotic cells in the brain tissues of neonatal mice in TLR3^−/−^+SEV group were all obviously reduced, indicating that TLR3 deletion improves the cell apoptosis in the brain tissues of neonatal mice with sevoflurane-induced cognitive dysfunction. The results of the *in vitro* experiment manifested that there were no obvious differences in the expressions of SHP2, p-RIP3, CaMKII, NLRP3, GSDMD, Caspase-1, Caspase-4 and Caspase-11 between RIP3^−/−^+SEV group and TLR3^−/−^+SEV group, suggesting that inhibiting the phosphorylation of RIP3 and repressing the expression of TLR3 exert the same effect on the expressions of these proteins in the hippocampus of neonatal mice with sevoflurane-induced cognitive dysfunction. The above results prompted that TLR3 influences neonatal mice with sevoflurane-induced cognitive dysfunction probably by regulating the phosphorylation of RIP3.

## CONCLUSIONS

Our data indicate that exposure to sevoflurane induces significant cognitive dysfunction, and that TLR3 deletion can prevent such neurological deficit in neonatal mice. The underlying neuroprotective mechanisms may involve inhibition of RIP3 phosphorylation and suppression of programmed necrosis of hippocampal neurons.
